# The comfort of patients with different nasal packings after endoscopic sinus surgery for chronic rhinosinusitis: A protocol for network meta-analysis

**DOI:** 10.1097/MD.0000000000016007

**Published:** 2019-06-14

**Authors:** Fuhong Zhang, Ji Chen, Xunwen Lei, Xiaowan Chen, Xiaobing Zhang

**Affiliations:** a Department of Otolaryngology Head and Neck Surgery, First Hospital of Lanzhou University; b The school of Nursing, Lanzhou university; c Department of Ophthalmology, First Hospital of Lanzhou University, Lanzhou, Gansu, People's Republic of China.

**Keywords:** chronic rhinosinusitis, endoscopic sinus surgery, nasal packing, network meta-analysis

## Abstract

**Background:**

Chronic sinusitis (CRS) is a high incidence disease and seriously affects the patients’ quality of life, causes tremendous economic burden as well. Endoscopic sinus surgery (ESS) is one of the most common therapies for chronic sinusitis. While whether there is a need for nasal packing and which is the best one is still unclear.

**Method and analysis:**

A systematic search will be performed using PubMed, EMBASE.com, the Cochrane Central Register of Controlled Trials, Chinese Biomedical Literature Database, China National Knowledge Infrastructure and Wanfang database to include randomized controlled trials (RCTs), comparing the efficacy and comfort in patients with nasal packings after ESS who is diagnosed as CRS. The risk of bias of the included RCTs will be assessed by the Cochrane Handbook version 5.1.0. A Bayesian network meta-analysis will be conducted using R-3.3.2 software.

**Results:**

This study is ongoing and the results will be published in a peer-reviewed journal.

**Conclusion:**

The results of this study will be sent to clinicians and healthcare providers in the National Health Service, which is expected to help clinicians make more informed choices on nasal packings.

**Ethics and dissemination:**

Ethical approval is not applicable since this study is a network meta-analysis based on published trials.

**Protocol registration number:**

CRD42019119233.

## 1 Introduction

Chronic sinusitis (CRS) is a high incidence disease characterized by pus, nasal obstruction, olfactory disturbance, headache, and other symptoms, lasting for more than 12 weeks, with severe cases having ocular compression and visual impairment, which can cause cranial, eye and lung complications. Chronic sinusitis is a high-risk disease. A multi-center questionnaire in China showed that the prevalence of CRS was 8% (4.8–9.7%).^[1]^ Meanwhile, CRS seriously affects the quality of life of 16% of American adults and 10.9% of European adults.^[2]^ Patients with chronic sinusitis reported more days in bed and more visits to family doctors, alternative health care providers and mental health specialists.^[3]^ At the same time, CRS causes a tremendous economic burden on the patients. A US survey shows that about $8.3 billion is spent annually on CRS treatment, with most being used for prescription drugs.^[4]^ Compared with congestive heart failure, coronary heart disease and chronic obstructive pulmonary disease, CRS patients scored significantly lower in terms of physical pain^[5]^ and social function, and the efficacy score was also poor.^[6]^

Endoscopic sinus surgery (ESS) has become the golden standard for CRS surgery.^[7]^ Nearly 250,000 ESS operations are carried out in the United States^[8]^ and about 40,000 in the United Kingdom^[9]^ each year. At present, it is still controversial whether nasal packing is necessary after ESS. But in most cases, nasal packings are used to alleviate bleeding, prevent adhesion and stabilize the nasal structure. There are many kinds of packing materials, including traditional Vaseline gauze and new material gelatin sponge, absorbable hemostatic gauze, expansive sponge, Nasopore, Rinogel, etc. Patients with nasal packings after ESS usually have different degrees of bleeding, exudation, headache, respiratory obstruction and other symptoms. Removal of nasal packing may cause re-injury to the nasal mucosa. And how to choose nasal packings becomes a clinical problem faced by doctors and patients since the price of materials varies greatly. In addition, nasal pain, frontal pain, orbital swelling pain and eye pain conjunctival congestions and swelling can be caused in the early stage after ESS because of the deep filling site.

At present, the clinicians have reached a consensus that CRS is a mixture of sinusitis with different pathogenesis, clinical manifestations and prognosis, which can be divided into different phenotypes and intrinsic types.^[10]^ Experts in this field have developed a series of guidelines and opinion papers on the diagnosis, classification and treatment of CRS, including perioperative management. However, there is no recommendation on the need for nasal packing and selection of nasal packings after the operation.

Network meta-analyses (NMAs) aim to rank the benefits (or harms) of interventions, based on all available randomized controlled trials.^[11]^ In this study, NMA will be used to evaluate the efficacy and comfort of nasal packings and to provide a guide for clinicians and patients to choose nasal packings.

## 2 Method

### 2.1 Design and registration

A network meta-analysis will be conducted to evaluate the effect and comfort of nasal packings after ESS in patients with CRS. This protocol has been registered on the international prospective register of a systematic review (PROSPERO), registration number: CRD42019119233. (https://www.crd.york.ac.uk/PROSPERO/#myprospero). No ethical approval is required since this study used data already in the public domain.

### 2.2 Inclusion criteria

#### 2.2.1 Types of study

All relevant RCTs will be included. Quasi-randomized trials will be excluded.

#### 2.2.2 Types of participants

Patients undergoing ESS for CRS will be included, and those with coagulation dysfunction and serious systemic diseases will be excluded. We will not consider the simultaneous endoscopic surgery for nasal septum, turbinate and nasal sinus tumors, nor do recurrent sinusitis and endoscopic sinus adhesions.

#### 2.2.3 Interventions

Nasal packing or none nasal packings after ESS.

#### 2.2.4 Types of outcomes

Primary outcomes: symptoms associated with nasal packings like bleeding with in situ packing, bleeding at removal, pain in situ, and nasal blockage, post-operative pain, edema, synechia/adhesion, and/or bleeding/hemostasis.

Secondary outcomes: recovery of nasal mucosa like mucosal edema, synechiae, infection and granulation.

## 3 Data sources and search strategies

### 3.1 Electronic databases

A systematic search will be performed using PubMed, EMBASE, the Cochrane Central Register of Controlled Trials (CENTRAL), the Chinese Biomedical Literature Database (CBM), China National Knowledge Infrastructure and Wanfang database will be searched.

### 3.2 Other sources

We will track the reference lists of included studies and check the existed systematic reviews to find relevant studies.

### 3.3 Search strategies

The search strategy was designed by ∗∗∗ and reviewed by ∗∗∗, who has been an information specialist for more than 10 years. And the searching of the database will be performed in January 2019, without language limitation. Parts of the strategies are listed in Table 1.

**Table 1:**
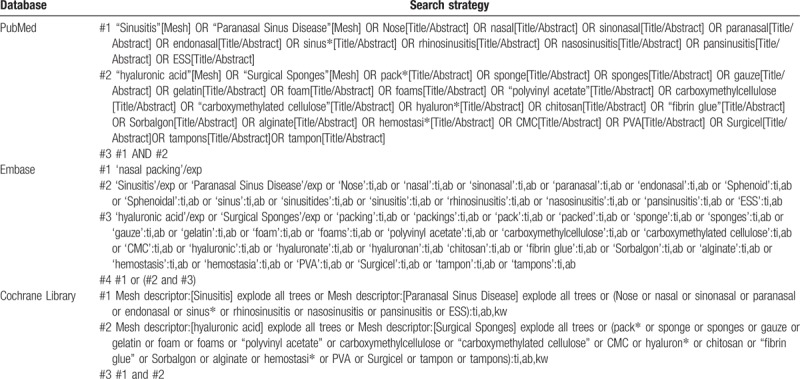
Search strategies.

### 3.4 Study identification

Citations of all identified systematic reviews will be downloaded to an EndNote database and then exported to a Microsoft Excel spreadsheet.^[12]^ The duplicated and apparently irrelevant studies will be excluded through titles and abstracts screening by two reviewers independently, and full texts of potentially eligible articles that will be assessed for inclusion independently by 2 reviewers.^[13]^ Any discrepancy in the selection of literature will be resolved by consensus or the third author.^[14]^

### 3.5 Data extraction

One reviewer will extract data from the included studies using a pre-compiled Excel^[15]^ form consisting of basic information of including studies, information about research design, characteristics of patients, interventions, participants’ outcomes, and another reviewer will check the extracted data. Any conflicts will be resolved by the discussion.^[16]^

Basic information of including studies: title, publication time, the first author, country.

Information about study design: sample size, random allocation, study period, study arms.

Characteristics of patients: year, sex, race, classification of CRS.

Interventions: types of nasal packings, length of insertion or the time to remove the nasal packings.

Participants’ outcomes: the primary outcomes and the secondary outcomes mentioned above and the duration of follow-up.

### 3.6 Methodological quality appraisal

Two reviewers will assess the methodological quality by the Cochrane “Risk of Bias” tool and grade the “risk of bias” as low, high, or unclear including random sequence generation (selection bias), allocation concealment (selection bias), blinding of participants and personnel (performance bias), blinding of outcome assessors (detection bias), incomplete outcome data (attrition bias), selective outcome reporting (reporting bias) and other potential sources of bias (e.g., design-specific risks of bias; baseline imbalance; differential diagnostic activity; contamination; fraud).^[17]^

### 3.7 Grade of evidence

Two authors will use the GRADE (Grading of Recommendations Assessment, Development and Evaluation) approach to evaluate the quality of evidence for each outcome, which is a tool to rate the quality of evidence of meta-analyses (MAs) and other bodies of evidence^[18]^ and the quality of evidence will be classified into four levels: high quality, moderate quality, low quality and very low quality. The quality rating of RCT may be rated down by −1 (serious concern) or −2 (very serious concern) for the following reasons: risk of bias, inconsistency, indirectness, imprecision and publication bias.^[19]^

### 3.8 Statistical analysis

We will analyze the relative outcomes of different nasal packings in patients with CRS after ESS from all direct and indirect comparisons and estimate the rank probabilities of all the groups using a Bayesian framework (White 2011; Higgins 2009).

### 3.9 Measures of treatment effect

For each dichotomous outcome, we will use risk ratios (RR) with 95% confidence intervals (CI) as a measure of treatment effect. For continuous variables, we will use the weighted mean difference (WMD) or standardized mean difference (SMD) with 95%CI for treatment effect measures, when they are measured using different scales.

### 3.10 Network meta-analysis

NMA will be conducted to explore the highest probability of being the most effective form of nasal packings by using R-3.3.2 software. Firstly, pairwise meta-analysis will be performed to synthesize results from head to head comparison between nasal packings under random effect model.^[20]^ I-square (I^2^) values will be calculated for quantifying heterogeneity among RCTs. The I^2^ value of <25%, 26% to 50%, >50% were regarded as low, moderate, and high heterogeneity respectively.^[21]^ And then indirect comparisons of the effectiveness among treatments will be conducted. The assumption of NMA was checked by evaluating inconsistency of direct and various indirect effect estimates, using the node splitting method for the same comparison. We will also assess the global heterogeneity on the bias of the magnitude of heterogeneity variance parameter (I2 or τ2 estimated from the network meta-analysis models using the *mtc.anohe* command of the ‘*gemtc’* package.^[22]^ To rank the efficacy for all treatments, we will use the Surface Under the Cumulative Ranking (SUCRA) values, and larger SUCRA values will indicate greater efficacy. Publication bias will be examined with the Begg^[23]^ funnel plot method.

## 4 Discussion

Compared with traditional surgery, there would be clearer vision and less trauma in ESS, aiming to restore the normal physiological structure and function of nasal cavity and paranasal sinus by removing irreversible local lesions. However, there still exist the post-operative wounds and possible bleeding. Clinicians should consider the safety and comfort of nasal packings after ESS. To our best knowledge, there are no NMAs comparing the comfort of patients with different nasal packings after ESS for CRS. This study will summarize the direct and indirect evidence, hoping to help clinicians and caregivers make more appropriate choices.

## Author contributions

FHC and JC planned and designed the study. XWL and XWC tested the feasibility of the study. FHC and JC provided methodological advice, considered for ideas and overall structure of the article and revised the manuscript. JC and XBZ wrote the manuscript. All authors approved the final version of the manuscript.

**Data curation:** Xiaowan Chen.

**Formal analysis:** Xiaowan Chen.

**Methodology:** Fuhong Zhang, Ji Chen.

**Project administration:** Fuhong Zhang.

**Resources:** Xunwen Lei.

**Software:** Xunwen Lei, Xiaobing Zhang.

**Writing – original draft:** Ji Chen, Xiaobing Zhang.
